# Establishment of epidemiological cutoff values against *Purpureocillium lilacinum*

**DOI:** 10.1128/jcm.00793-26

**Published:** 2026-08-21

**Authors:** Dallas J. Smith, Marisa L. Winkler, Philippe J. Dufresne, Warda Memon, Julianne V. Kus, Lisa McTaggart, Yalong Li, Rodrigo Almeida-Paes, Gloria M. González, Huan Mei, Ruoyu Li, Jasmijn Jansen, Yinggai Song, Jos Houbraken, Ferry Hagen, Amir Seyedmousavi, Sean X. Zhang, Mariana Castanheira, Shawn R. Lockhart, Jochem B. Buil, Andrew M. Borman, Nathan P. Wiederhold

**Affiliations:** 1 Mycotic Diseases Branch, Centers for Disease Control and Prevention 1242, Atlanta, Georgia, USA; 2 Element Iowa City (JMI Laboratories) 138461, North Liberty, Iowa, USA; 3 Laboratoire de santé publique du Québec, Institut national de santé publique du Québec (INSPQ) 54470, Québec, Canada; 4 Microbiology Laboratory, Johns Hopkins Hospital 588543, Baltimore, Maryland, USA; 5 Public Health Ontario 153300, Toronto, Ontario, Canada; 6 Department of Laboratory Medicine and Pathobiology, University of Toronto 7938, Toronto, Ontario, Canada; 7 Department of Dermatology and Venerology, Peking University First Hospital 26447, Beijing, China; 8 Research Center for Medical Mycology, Peking University 12465, Beijing, China; 9 National Clinical Research Center for Skin and Immune Diseases, Beijing, China; 10 Beijing Key Laboratory of Molecular Diagnosis on Dermatoses, Beijing, China; 11 Laboratório de Micologia, Instituto Nacional de Infectologia Evandro Chagas, Fundação Oswaldo Cruz, Fiocruz 663758 , Rio de Janeiro, Brazil; 12 Departamento de Microbiología, Facultad de Medicina, Universidad Autónoma de Nuevo León 27771, Monterrey, Nuevo León, Mexico; 13 Department of Medical Mycology, Hospital for Skin Diseases, Institute of Dermatology, Chinese Academy of Medical Sciences & Peking Union Medical College, Nanjing, China; 14 Department of Medical Microbiology, Radboud University Medical Center 6034, Nijmegen, the Netherlands; 15 Department of Medical Microbiology, Radboudumc-Canisius Wilhelmina Ziekenhuis Center of Expertise for Mycology, Nijmegen, the Netherlands; 16 Westerdijk Fungal Biodiversity Institute 141042, Utrecht, the Netherlands; 17 Department of Medical Microbiology, University Medical Center Utrecht, Utrecht, the Netherlands; 18 Microbiology Service, National Institutes of Health Clinical Center 24481, Bethesda, Maryland, USA; 19 Department of Pathology, Johns Hopkins University School of Medicine 1500, Baltimore, Maryland, USA; 20 UK HAS National Mycology Reference Laboratory, Science Quarter, Southmead Hospital 159003, Bristol, United Kingdom; 21 Medical Research Council Centre for Medical Mycology (MRC CMM), University of Exeter 3286, Exeter, United Kingdom; 22 The University of Texas Health Science Center 14742, San Antonio, Texas, USA; University of Utah Health Medicine, Salt Lake City, Utah, USA

**Keywords:** epidemiological cutoff values, antifungal resistance, hyalohyphomycosis, *Purpureocillium lilacinum*

## Abstract

*Purpureocillium lilacinum*, a common environmental fungus, can cause hyalohyphomycosis with varied clinical manifestations. Mortality rates can reach 20% with invasive disease. *P. lilacinum* is intrinsically resistant to amphotericin B, leaving few treatment options. In the absence of clinical breakpoints, epidemiological cutoff values (ECVs) can aid clinicians in monitoring antifungal resistance trends and in guiding initial therapy. We performed antifungal susceptibility testing (AFST) on *P. lilacinum* isolates to establish minimum inhibitory concentration (MIC) and minimum effective concentration (MEC) distributions and ECVs. Data were collected from 13 laboratories located in seven countries. AFST was performed by broth microdilution (CLSI M38 standard) for itraconazole, voriconazole, posaconazole, isavuconazole, terbinafine, flucytosine, amphotericin B, natamycin, and manogepix. ECVs were established using the iterative statistical method with ECOFFinder (v2.1) following CLSI M57 guidelines. Results from 479 *P. lilacinum* isolates were analyzed. ECVs were 1 μg/mL for voriconazole, 2 μg/mL for posaconazole, 4 μg/mL for isavuconazole, and 2 μg/mL for terbinafine. Itraconazole displayed a trimodal MIC distribution, although almost all MICs were ≥0.5 µg/mL (mode, 16 μg/mL). Amphotericin B, natamycin, and flucytosine MICs were truncated at the high end of the range and consistent with intrinsic resistance. The manogepix modal MEC was 0.008 μg/mL. These proposed *P. lilacinum* ECVs and MIC/MEC distributions provide baseline data to monitor resistance trends. Our results confirm that *P. lilacinum* is intrinsically resistant (IR) to amphotericin B and likely IR to natamycin and flucytosine. Caution is needed in interpreting itraconazole results due to high interlaboratory variability.

**IMPORTANCE:**

*Purpureocillium lilacinum* is a hyaline mold commonly found in the environment and used as an agricultural biopesticide. Infections caused by *P. lilacinum* can range from fungal eye infections to life-threatening pulmonary and disseminated disease, and mortality rates can be high (up to 20%). Single-center studies examining antifungal susceptibility testing (AFST) data found reduced susceptibility to certain antifungals, but more robust global AFST studies are needed to help interpret MICs and MECs. We performed AFST according to the Clinical Laboratory and Standards Institute reference method in thirteen independent laboratories on 479 *P. lilacinum* isolates to establish MIC/MEC distributions and epidemiological cutoff values (ECVs) for nine antifungals, including those recommended by global treatment guidelines. The calculated ECVs were 1 μg/mL for voriconazole, 2 μg/mL for posaconazole, 4 μg/mL for isavuconazole, and 2 μg/mL for terbinafine. Our results also confirm that *P. lilacinum* is intrinsically resistant to amphotericin B and likely intrinsically resistant to natamycin and flucytosine. Caution is needed in interpreting itraconazole AFST results due to high interlaboratory variability. These antifungal *in vitro* susceptibility data suggest limited options to treat *P. lilacinum* infections.

## INTRODUCTION

*Purpureocillium lilacinum* (formerly *Paecilomyces lilacinus*) is a hyaline mold commonly found in the environment. The fungus has been found in soil, decaying vegetation, insects, nematodes, water streams, hospital water supply systems, and in laboratories as a contaminant (1–5). Various strains of *P. lilacinum* are approved and used as agricultural bionematicides globally, including in the United States (6–8).

In humans, *P. lilacinum* causes hyalohyphomycosis with varied clinical presentations. The most commonly reported clinical manifestations are eye infections (e.g., fungal keratitis) (9, 10). Other invasive infection sites are the skin, lungs, deep soft tissue, bloodstream, and sinuses; dissemination can occur and adjacent organs can become involved (11). Common risk factors for *P. lilacinum* infections include hematological and oncological diseases, use of steroids, solid organ transplantation, and diabetes (11), although disease can also occur in immunocompetent patients (12). Outbreaks have been linked to gaps in infection prevention and control in eye clinics and from contaminated skin lotion (5, 13).

The identification of *P. lilacinum* by histopathology in tissue is challenging, as morphology is indistinguishable from *Aspergillus* and other hyaline molds. Often, *P. lilacinum* is confirmed by fungal culture, with a median time from collection to result of 23 days (14). *P. lilacinum* isolates can be identified morphologically based on their characteristic pink- to light-purple- or lilac-colored colonies and their long tapered penicillium-like phialides. Definitive species identification can be performed by molecular sequencing of the internal transcribed spacer (ITS) barcode or matrix-assisted laser desorption ionization-time-of-flight mass spectrometry (MALDI-TOF MS) (15). Distinguishing between *P. lilacinum* and morphologically similar *Paecilomyces* species is particularly important, as susceptibility profiles and treatment options differ (10).

Multicenter antifungal susceptibility testing (AFST) studies using Clinical Laboratory and Standards Institute (CLSI) methodology for *P. lilacinum* are lacking. Data from the United Kingdom Mycology Reference Laboratory showed modal minimum inhibition concentration (MIC) of 16 μg/mL for amphotericin B, 0.5 μg/mL for itraconazole, 0.25 μg/mL for posaconazole, and 0.125 μg/mL for voriconazole (16). CLSI has designated *P. lilacinum* as intrinsically resistant to amphotericin B based on *in vitro* AFST data (17). The use of *P. lilacinum* in agriculture and the impact of environmental pressure on susceptibility profiles is unknown. For example, spraying *P. lilacinum* on agricultural fields that are also sprayed with fungicides could increase selective pressure for isolates with higher MICs, which has been observed with *Aspergillus fumigatus* in the environment (18).

The optimal treatment for infections caused by *P. lilacinum* is unknown and may differ depending on clinical presentation. The European Confederation of Medical Mycology, International Society for Human and Animal Mycology, and the American Society for Microbiology rare mold infections guidelines moderately recommend voriconazole first-line and marginally recommend itraconazole, posaconazole, or liposomal amphotericin B as second-line or as salvage treatment (19). For cutaneous or subcutaneous infections, the guidelines moderately recommend voriconazole plus terbinafine as first-line therapy. A retrospective study of clinical infections found a significantly higher mortality rate in patients treated with amphotericin B compared with those receiving systemic treatment without amphotericin B (11).

Breakpoints for *P. lilacinum* have not been established, making it difficult to interpret an MIC for most antifungal agents. In the absence of breakpoints, epidemiological cutoff values (ECVs) can predict if a fungal isolate is non-wild type in regard to its *in vitro* response to an antifungal (20). ECVs can be useful to monitor changes in antifungal resistance due to environmental pressures or selection. ECVs are not intended to replace clinical breakpoints, as they are based solely on the *in vitro* MIC distribution of a population and do not incorporate clinical outcome data or pharmacokinetic and pharmacodynamic parameters. However, ECVs may help inform treatment decisions, as non-wild type strains may not respond to a specific antifungal therapy the same way as wild-type strains of the same species. Consequently, when a non-wild type strain is identified, an alternative antifungal, if available, should be considered for treatment. Few molds, including hyaline molds, have CLSI-approved ECVs (20, 21). Thus, we performed AFST on *P. lilacinum* isolates to establish MIC distributions, ECVs, percentage non-wild type, and modal MICs/MECs for nine antifungals: itraconazole, voriconazole, posaconazole, isavuconazole, terbinafine, flucytosine, amphotericin B, natamycin, and manogepix (the active moiety of the prodrug fosmanogepix).

## RESULTS

AFST was performed by broth microdilution (BMD) as outlined in the CLSI reference standard M38 Ed3 for filamentous fungi (22). Four hundred and seventy-nine *P. lilacinum* isolates originating from 24 different countries were tested in thirteen different laboratories (Table 1). ECVs were determined using the iterative statistical method with ECOFFinder (V2.1) with a 97.5% threshold following CLSI M57 guidelines (20, 23). The calculated ECVs were 1 μg/mL for voriconazole, 2 μg/mL for posaconazole, 4 μg/mL for isavuconazole, and 2 μg/mL for terbinafine (Table 2). Itraconazole had a trimodal MIC distribution; therefore, no ECV was set. However, almost all itraconazole MICs were elevated (≥0.5 µg/mL). Amphotericin B (Fig. S1), natamycin (Table S1), and flucytosine MIC profiles were truncated on the high end of the concentration range tested. No ECV was set for manogepix due to insufficient data.

**TABLE 1:** Minimum inhibitory concentrations for *Purpureocillium lilacinum* and seven antifungals^*a*^

Antifungal (no. of isolates)^a^	No. of isolates with MIC (μg/mL) of:											
	≤0.016	0.03	0.06	0.12	0.25	0.5	1	2	4	8	16	≥32
Itraconazole (395 from 13 labs)		1	0	1	9	50	42	63	46	12	**92**	79
Voriconazole (479 from 13 labs)		2	10	67	**179**	156	50	12	2	1		
Posaconazole (356 from 13 labs)		1	8	34	82	**131**	90	6	3	1		
Isavuconazole (231 from 13 labs)			1	4	23	54	**80**	48	19	1		1
Terbinafine (131 from 7 labs)	2	4	26	30	29	**31**	7	2				
Flucytosine (133 from 7 labs)									1			**132**
Amphotericin B (438 from 13 labs)						1		6	3	16	111	**301**

^*a*^ Some laboratories were unable to perform antifungal susceptibility testing on all antifungals due to inability to test (not routinely tested) an antifungal or difficulties with a *P. lilacinum* isolate. Modal MICs are in bold.

**TABLE 2:** Epidemiological cutoff values and minimum inhibitory concentration characteristics for *Purpureocillium lilacinum* and seven antifungals^*c*^

Antifungal	ECV (μg/mL)	% NWT	Modal MIC (μg/mL)
Itraconazole	N/A^*a*^	N/A	16
Voriconazole	1	3%	0.25
Posaconazole	2	1%	0.5
Isavuconazole	4	1%	1
Terbinafine	2	0%	0.5
Flucytosine	TR-H	N/A	32
Amphotericin B	TR-H (intrinsically resistant)^*b*^	N/A	32

^*a*^ Itraconazole displayed trimodal distribution and strong intra- and inter-lab variation for *Purpureocillium lilacinum*. A reliable epidemiological cutoff value could not be set.

^*b*^ Stated as intrinsically resistant in Clinical Laboratory and Standards Institute M38M51S.

^*c*^ ECV, epidemiological cutoff value; NWT, non-wild type; MIC, minimum inhibitory concentration; N/A, not applicable; TR-H; MIC distribution is truncated on high end of testing range.

The percentage of non-wild type isolates (MICs greater than the established ECV) was 3% (15/479) for voriconazole, 1% (4/356) for posaconazole, 1% (2/231) for isavuconazole, and 0% (0/131) for terbinafine (Table 2). Modal MICs were 16 μg/mL for itraconazole, 0.25 μg/mL for voriconazole, 0.5 μg/mL for posaconazole, 1 μg/mL for isavuconazole, 0.5 μg/mL for terbinafine, 32 μg/mL for flucytosine, 32 μg/mL for amphotericin B, >32 µg/mL for natamycin, and the modal MEC was 0.008 μg/mL for manogepix (Table S2).

## DISCUSSION

Using CLSI BMD methodology, we established ECVs for *P. lilacinum* for guideline-recommended first- and second-line antifungals, as well as those used as salvage therapy, to assist clinicians in making treatment decisions and to assist public health professionals to monitor for antifungal resistance. A global network of thirteen laboratories in seven countries contributed data on 479 isolates from 24 countries, which strengthens the applicability of these results. Invasive *P. lilacinum* infections carry high mortality rates, and these ECVs can assist with selecting appropriate antifungal therapy (11). Environmental pressures on susceptibility profiles can also be monitored via these ECVs.

Our data provide robust support for designating *P. lilacinum* as intrinsically resistant to amphotericin B. Combined with clinical evidence that outcomes are poorer in infections treated with amphotericin B, clinicians may choose to seek alternative treatment (11). Updates to the global guideline for the diagnosis and management of rare mold infections may be warranted to remove the marginal recommendation for amphotericin B use as second-line or salvage monotherapy (19). Similar to amphotericin B, our MICs for the polyene natamycin were very high against *P. lilacinum*, suggesting intrinsic resistance. Additionally, clinicians and clinical microbiologists should be cautious in interpreting itraconazole MICs as high interlaboratory variability was observed. The reason for this variability is unknown, but may be related to poor itraconazole aqueous solubility, resulting in its precipitation out of solution at higher concentrations, or possibly to an efflux pump mechanism (24). Paradoxical growth at concentrations above the MIC (Eagle-like effect) may also be observed with itraconazole for some molds, which could render MIC reading difficult if it occurs for *P. lilacinum*. This may result in clinical dilemmas as the rare mold guidelines marginally recommend itraconazole for second-line or salvage therapy (19). The ECV for terbinafine (2 μg/mL) is high, and this antifungal may not be appropriate as monotherapy against *P. lilacinum* infections. Interestingly, the rare mold guidelines recommend a combination of voriconazole and terbinafine, with or without surgery, as first-line therapy for cutaneous and subcutaneous infections by *P. lilacinum* (19). However, this recommendation is based on case reports, which demonstrated successful outcomes with this approach, without direct comparison with voriconazole monotherapy. Consequently, the contribution of terbinafine to treatment success remains unclear (25, 26). An *in vitro* synergistic effect between voriconazole and terbinafine has been noted against other molds, such as *Lomentospora prolificans*, but not specifically in *P. lilacinum* (27–29). Finally, *P. lilacinum* is likely intrinsically resistant to flucytosine (modal MIC of 32 μg/mL). Given the high *in vitro* MICs observed, this antifungal should be avoided as monotherapy for the treatment of *P. lilacinum* infections, and its role in combination therapy for refractory infections is questionable.

In *P. lilacinum* keratitis, treatment options are few with intrinsic resistance to amphotericin B and most likely natamycin. A randomized clinical controlled trial found no benefit of oral voriconazole in treating fungal keratitis caused by filamentous fungi, although this study did not include *P. lilacinum* infections (30). These data suggest that topical voriconazole may be the only appropriate option for *P. lilacinum* keratitis, highlighting the need for novel antifungal therapies with good penetration into the eye. Based on these *in vitro* data, fosmanogepix may be a promising option for both keratitis and invasive infections caused by *P. lilacinum*, but additional data, including those from pharmacodynamic/pharmacokinetic and clinical studies, will be critical to assess its effectiveness (31).

Although *P. lilacinum* is used globally as a biopesticide in the environment, these *in vitro* AFST data suggest that the fungus has not yet acquired resistance more broadly, unlike what is being seen with *Aspergillus fumigatus* and triazole antifungals (18). The prevalence of non-wild type isolates ranged from 1%–3% for antifungals with an established ECV. Acquired antifungal resistance has been reported in an agricultural worker with endophthalmitis caused by *P. lilacinum* (32). Interestingly, in our study, one isolate had an MIC of ≥16 µg/mL for isavuconazole. Although clinical data were not available for this isolate, the high MIC raises concern for microbiological resistance. Additionally, using these ECVs to identify non-wild type isolates may serve as early warning indicators for environmentally acquired phenotypic changes in isolates. Further investigation into intrinsic resistance mechanisms for amphotericin B and flucytosine, along with whole-genome sequencing of non-wild type isolates for other antifungals, can help elucidate *P. lilacinum* antifungal resistance mechanisms.

The interpretation of these data and ECVs is subject to several limitations. First, clinical data were unavailable, so we were unable to correlate *in vitro* findings to patient outcomes. MIC results alone are not necessarily predictive of clinical success or failure, as other factors, including host immune status and other treatment modalities, can positively or negatively influence patient outcomes. Second, although this is the largest collection of AFST data for *P. lilacinum*, these data are a convenience sample from laboratories with the capacity to identify and perform AFST on this fungus and may not represent global susceptibility profiles. Third, the MIC data were gathered retrospectively in most laboratories over many years, and the variability observed could be due to changes in susceptibility testing reagents and practices during that time period.

### Conclusion

These proposed ECVs and MIC/MEC distributions, obtained through a multicenter international effort, provide baseline data for *P. lilacinum* to monitor antifungal resistance resulting from environmental (e.g., use as a pesticide) pressure. Our results also confirm that *P. lilacinum* is intrinsically resistant to amphotericin B and likely also to natamycin and flucytosine. Caution is needed in interpreting itraconazole AFST results with high interlaboratory variability, and more work is warranted to understand the source of this variation, given that all laboratories utilized CLSI M38 reference broth microdilution methodology. These *in vitro* data suggest limited options to treat *P. lilacinum* infections, although novel antifungals like manogepix show good *in vitro* activity.

## MATERIALS AND METHODS

### Isolates

Species identification was confirmed by DNA analysis using ITS sequencing or MALDI-TOF MS. Among the 479 *P. lilacinum* isolates, 212 came from the United States, 125 from the United Kingdom, 55 from the Netherlands, 33 from Canada, 20 from China, four from Brazil, Mexico, and Slovenia, three from New Zealand, two from Chile, Germany, Portugal, and Thailand, and one each from Australia, the Bahamas, Colombia, France, Hungary, Israel, Kuwait, Japan, Ireland, Sweden, and Turkey. The countries in which the isolates were tested do not necessarily represent the countries where they were collected.

Isolate data were submitted by thirteen different laboratories on four continents: 153 from Fungus Testing Laboratory, University of Texas Health Science Center, San Antonio, Texas, USA; 121 from UK Health Security Agency, National Mycology Reference Laboratory, Bristol, UK; 47 from Department of Medical Microbiology, Radboudumc, Nijmegen, The Netherlands; 40 from Element Iowa City (JMI Laboratories), North Liberty, IA, USA; 30 from Westerdijk Fungal Biodiversity Institute (WI-KNAW), Utrecht, the Netherlands; 23 from Laboratoire de santé publique du Québec, Québec, Canada; 19 from Department of Pathology, Johns Hopkins University School of Medicine, Baltimore, MD, USA; 10 from Research Center for Medical Mycology, Peking University, Beijing, China; ten from Public Health Ontario, Toronto, Ontario, Canada; nine from Department of Medical Mycology, Institute of Dermatology, Chinese Academy of Medical Sciences and Peking Union Medical College, Nanjing, China; nine from Department of Laboratory Medicine, National Institute of Health, Bethesda, MD, USA; four from Laboratório de Micologia, Instituto Nacional de Infectologia Evandro Chagas, Fundação Oswaldo Cruz, Fiocruz, Rio de Janeiro, RJ, Brazil; and four from Departamento de Microbiología, Facultad de Medicina, Universidad Autónoma de Nuevo León, Monterrey, Nuevo León, Mexico.

### Antifungal susceptibility testing

Four hundred and seventy-nine *P. lilacinum* isolates were used in this study. AFST was performed by broth microdilution as outlined in the CLSI reference standard M38 Ed3 for filamentous fungi (22). Isolates used as reference strains or quality control strains were *Aspergillus fumigatus* ATCC MYA-3626, *Aspergillus flavus* ATCC 204304*, Candida parapsilosis* ATCC 22019 (=CBS 604), *Pichia kudriavzevii* ATCC 6258 (=CBS 573), and *Hamigera insecticola* ATCC MYA-3630; quality control values were within M38M51S CLSI ranges (17). Itraconazole, voriconazole, posaconazole, isavuconazole, terbinafine, flucytosine, amphotericin B, natamycin, and manogepix were tested. The MICs were determined visually after 24–48 h (1–2 days, depending on the growth rate of the individual isolate) of incubation at 35°C. Inoculum was adjusted with an absorbance 530 nm at 0.15–0.17 and verified by hematocytometer counting as within 0.2 to 2.5 × 10^6^ conidia/mL or by Cellometer X2 (Nexcelom, Manchester, United Kingdom) to perform cell counts and used that to prepare the inoculum that was within the range for further processing. The antifungal susceptibility endpoints for itraconazole, voriconazole, posaconazole, isavuconazole, terbinafine, natamycin, and amphotericin B were 100% inhibition of growth. For flucytosine, ≥50% inhibition of growth compared to the positive control was used. For manogepix, the MECs were measured and defined as the lowest concentration resulting in a morphological change to a compact, rounded, granular form with no long hyphae visible.

### Epidemiological cutoff values

The ECVs were established using the iterative statistical method with ECOFFinder (V2.1) with a 97.5% threshold following CLSI M57 guidelines (20, 23). Percentage of non-wild type isolates and modal MICs/MECs were calculated for each antifungal.

## SUPPLEMENTAL MATERIAL

Supplemental figure and tables jcm.00793-26-s0001.docx Fig. S1, and Tables S1 and S2.

ASM does not own the copyrights to Supplemental Material that may be linked to, or accessed through, an article. The authors have granted ASM a non-exclusive, world-wide license to publish the Supplemental Material files. Please contact the corresponding author directly for reuse.
