# Supplementary material for: Establishment of epidemiological cutoff values against Purpureocillium lilacinum
Source: J Clin Microbiol. 2026 Aug 21;64(9):e00793-26. doi: 10.1128/jcm.00793-26 (PMC13556260; doi:10.1128/jcm.00793-26)
Supplement: Supplementary file 1 [file jcm.00793-26-s0001.docx]

**Supplemental Figure 1: Epidemiological cutoff value finder demonstrating *Purpureocillium lilacinum* amphotericin B minimum inhibitory concentrations truncated at the high end of the testing range**

Count/Percentage

| [MIC] | Raw Count or % bar | Raw Count or % | Fitted Count or % |
| --- | --- | --- | --- |
| 0.000976562 |  |  | #N/A |
| 0.00195312 |  |  | #N/A |
| 0.00390625 |  |  | #N/A |
| 0.0078125 |  |  | #N/A |
| 0.015625 |  |  | #N/A |
| 0.03125 |  |  | #N/A |
| 0.0625 |  |  | #N/A |
| 0.125 |  |  | #N/A |
| 0.25 |  |  | #N/A |
| 0.5 | 1 | 1 | #N/A |
| 1 |  |  | #N/A |
| 2 | 6 | 6 | #N/A |
| 4 | 3 | 3 | #N/A |
| 8 | 16 | 16 | #N/A |
| 16 | 111 | 111 | #N/A |
| 32 | 301 | 301 | #N/A |
| 64 |  |  | #N/A |
| 128 |  |  | #N/A |
| 256 |  |  | #N/A |
| 512 |  |  | #N/A |
| 1024 |  |  | #N/A |

**Supplemental table 1: Natamycin minimum inhibitory concentrations against *Purpureocillium lilacinum***

| **Antifungal (number of isolates)*** | **Number of Isolates per Minimum Inhibitory Concentrations in μg/mL** | | | | | | | | | | | | |
| --- | --- | --- | --- | --- | --- | --- | --- | --- | --- | --- | --- | --- | --- |
|  | **0.03** | **0.06** | **0.12** | **0.25** | **0.5** | **1** | **2** | **4** | **8** | **16** | **32** | **>32** |  |
| **Natamycin (97)** |  |  |  |  |  |  | 1 | 2 | 1 | 1 | 2 | **90** |  |

*****Isolates were tested in two laboratories. Modal minimum inhibitory concentration is bold.

**Supplemental table 2: Manogepix minimum effective concentrations against *Purpureocillium lilacinum***

| **Antifungal (number of isolates)*** | **Number of Isolates per Minimum Effective Concentrations in μg/mL** | | | | | | | | | | | | |
| --- | --- | --- | --- | --- | --- | --- | --- | --- | --- | --- | --- | --- | --- |
|  | **0.002** | **0.004** | **0.008*** | **0.016** | **0.03** | **0.06** | **0.12** | **0.25** | **0.5** | **1** | **2** | **4** |  |
| **Manogepix (66)** |  | 17 | **26** | 14 | 5 | 3 | 1 |  |  |  |  |  |  |

*Four labs contributed minimum effective concentration (MEC) data. One lab did not test concentrations ≤0.008. Nineteen isolates in this category had MECs of ≤0.008. Modal MEC is bold.
